# *Mycobacterium sherrisii* Lung Infection in a Brazilian Patient with Silicosis and a History of Pulmonary Tuberculosis

**DOI:** 10.1155/2015/498608

**Published:** 2015-10-18

**Authors:** Carolina de Oliveira Abrão, João Alves de Araújo Filho

**Affiliations:** ^1^Clinics Hospital, Federal University of Goiás, 1^a^ Avenida, s/n, Setor Leste Universitário, 74605-020 Goiânia, GO, Brazil; ^2^Department of Tropical Medicine and Dermatology, Tropical Pathology and Public Health Institute, Federal University of Goiás, Goiânia, GO, Brazil; ^3^Hospital of Tropical Diseases Dr. Anuar Auad, Goiânia, GO, Brazil

## Abstract

Nontuberculous mycobacteria (NTM) diseases became relevant with the emergence and spread of HIV and are also related to lung infection in non-HIV individuals with structural lung diseases. *Mycobacterium sherrisii* is a NTM first characterized in 2004. Only a few cases have been reported. The aim of this case report is to describe the first detailed case of infection with *M. sherrisii* in a patient with silicosis and history of pulmonary tuberculosis. A 50-year-old HIV-negative white male, previous smoker, with silicosis and a history of treated pulmonary tuberculosis developed a worsening of cough and expectoration pattern, and two sputum samples were positive for acid-fast bacilli. Presumptive treatment for pulmonary tuberculosis was initiated with rifampin, isoniazid, pyrazinamide, and ethambutol, but, at month 5 of treatment, despite correct medication intake and slight improvement of symptoms, sputum bacilloscopy remained positive. Sputum cultures were positive *Mycobacterium sherrisii*. Treatment regimen was altered to streptomycin (for 2 months), ethambutol, clarithromycin, rifabutin, and trimethoprim-sulfamethoxazole. *M. sherrisii* should be considered a possible etiological agent of lung infections in patients with pneumoconiosis and history of tuberculosis.

## 1. Introduction

The genus* Mycobacterium* consists of a group of various acid-fast bacilli that can cause infections in humans and animals. In addition to the* Mycobacterium tuberculosis* complex and* Mycobacterium leprae*, it includes the organisms known as nontuberculous mycobacteria (NTM). The NTM are usually not pathogenic environmental microorganisms [1–3].

In immunocompetent patients, NTM can occasionally cause disease, mostly in patients with a preexisting pulmonary pathology. In severely immunocompromised patients, especially those with acquired immunodeficiency syndrome (AIDS), NTM infections can disseminate and result in morbidity and high mortality rates [1–3].

*Mycobacterium sherrisii* is a NTM closely related to* M. simiae*. It was first characterized in 2004 by Selvarangan et al., but it was only recognized as a novel species in 2011 [4, 5]. Since then, only a few cases have been reported, mainly in patients immunosuppressed by human immunodeficiency virus (HIV) [6–11], but also in patients with underlying pulmonary disease without HIV infection [12, 13]. However, in the latter, no cases in silicotic patients have been described in detail.

Thus, the aim of this case report is to describe the first detailed case of infection with* M. sherrisii* in a patient with silicosis and a previous history of pulmonary tuberculosis.

## 2. Case Presentation

The patient is a 50-year-old white male, previous smoker (he smoked for 30 years, 45 pack-years, quitting 8 years ago), who worked in an underground emerald mine for 20 years, until 9 years ago, without proper use of personal protective equipment. He had a history of pulmonary tuberculosis treated with rifampin, isoniazid, and pyrazinamide for 2 months, followed by rifampin and isoniazid for 4 months, 11 years ago.

He attends a pneumology outpatient clinic due to silicosis and, as of 2013, a high-resolution computed tomography of the thorax already showed structural lung sequelae.

**Figure 1:**
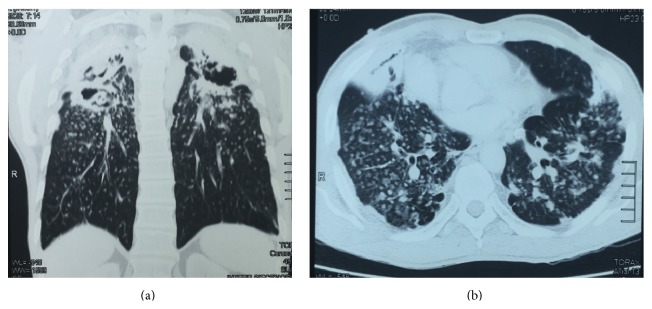
Thorax computed tomography: large pulmonary opacities in the upper lobes of both lungs, air bronchograms and calcifications, bilateral diffuse nodular infiltrates with areas of pleural thickening, nodular interstitial infiltrate associated with ground glass opacities, and cavitations with thick walls. (a) Coronal plane. (b) Axial plane.

During follow-up, he developed a worsening of cough and expectoration pattern. Two sputum samples were positive for acid-fast bacilli [(+) and (++), resp.], and presumptive treatment for pulmonary tuberculosis was initiated with rifampin, isoniazid, pyrazinamide, and ethambutol. Mycobacteria culture was not performed at this moment. However, at month 5 of treatment, already in the continuation phase with rifampin and isoniazid, and despite correct medication intake and a slight improvement of symptoms, sputum bacilloscopy remained positive. A thorax computed tomography revealed large pulmonary opacities in the upper lobes of both lungs, with intermingled air bronchograms and calcifications, bilateral diffuse nodular infiltrates with areas of pleural thickening, and nodular interstitial infiltrate associated with ground glass opacities in the posterior segment of the inferior right lobe, as well as cavitations with thick walls, suggesting an active inflammatory process (Figure 1). Blood tests revealed a normal hemogram and renal and liver functions, and HIV, hepatitis B, and hepatitis C serologies were negative.

Sputum cultures were then performed and two were positive for NTM; while awaiting the identification of the species, empiric treatment with streptomycin, ethambutol, clarithromycin, and rifampin was instituted for* Mycobacterium avium*, the most frequently NTM isolated in our community. The patient had a greater improvement of his symptoms.

The species identification was performed by the Tuberculosis Bacteriology Laboratory from the Professor Hélio Fraga Reference Center (Rio de Janeiro), the National Mycobacterium Reference Laboratory, using genetic sequencing of the 16S rRNA gene. The NTM species isolated was* Mycobacterium sherrisii*. The treatment regimen was once again adjusted, with the replacement of rifampin with rifabutin, inclusion of trimethoprim-sulfamethoxazole (TMP-SMX), and suspension of streptomycin after 2 months. A sensitivity test was not performed.

Currently, the patient is at month 9 of treatment, with only an occasional nonproductive cough, and with negative sputum bacilloscopies and cultures, which have been performed monthly, since month 2 of treatment.

It is our plan to complete 12 months of treatment with rifabutin, clarithromycin, ethambutol, and TMP-SMX, as of the first negative culture.

## 3. Discussion

Nontuberculous mycobacterial (NTM) diseases became relevant with the emergence and spread of infection by human immunodeficiency virus (HIV) and are also often related to lung disease in HIV-negative individuals [1–3], particularly those with structural lung diseases such as bronchiectasis, cystic fibrosis, pneumoconiosis, chronic obstructive pulmonary disease, pulmonary alveolar proteinosis, and history of tuberculosis, as well as esophageal motility disorders.

Advances in microbiology and molecular techniques, mainly in the 16S rRNA gene sequencing, led to the characterization of several new NTM species [1]. Currently, there are more than 160 of them (http://www.bacterio.net/mycobacterium.html).* Mycobacterium sherrisii* is one of those new species [4, 5]. In 2004, Selvarangan et al. [4], based on fatty acid analysis and genotypic evaluation of five mycobacterial isolates similar to* M. simiae*, proposed the characterization of* M. sherrisii* as a new species of the genus* Mycobacterium*. In 2011, Van Ingen et al. [5] further described this new species. In summary,* M. sherrisii* is a slow-growing (over 7 days), nonchromogenic mycobacteria. Phylogenetically, it is similar to* M. simiae* and* M. triplex*.

Since first being recognized as a species,* M. sherrisii* has been isolated and related to infections in both child and adult AIDS patients, mainly in Africa [6–8, 11, 14], but not exclusively [10, 13, 15], causing pulmonary or disseminated disease, including in the context of immune reconstitution inflammatory syndrome (IRIS) [9]. It was also isolated along with* Histoplasma capsulatum* in an African HIV patient [6].

*M. sherrisii* has also been implicated in infections in immunocompetent individuals. The first case in a non-HIV patient was described by Tortoli et al. [12], of a male with rheumatoid arthritis and lung infiltrates in the left upper lobe with homolateral pleural effusion. Barrera et al. [13] briefly described 3 cases of pulmonary infection with* M. sherrisii* in non-HIV patients. One of them had silicosis and two had pulmonary tuberculosis.

Our 50-year-old patient worked for several years in underground emerald mining, in the state of Goiás (Central-West region of Brazil), without proper use of personal protection equipment (PPE), which led to the development of pneumoconiosis. He also reported a previous episode of pulmonary tuberculosis in 2004. Both diseases are known predisposing factors for NTM lung infection.

Six months before consulting in our institution, treatment for pulmonary tuberculosis with rifampin, isoniazid, pyrazinamide, and ethambutol was started due to worsening of respiratory status, and the finding of acid-fast bacilli in sputum. However, since only partial clinical improvement was achieved, and the sputum remained persistently positive for acid-fast bacilli, cultures were requested and NTM was isolated, which was later identified as* M. sherrisii*. Detailed case reports on infection with these bacteria in individuals with pneumoconiosis and a history of tuberculosis were not found after searches on PubMed and LILACS using the uniterms pneumoconiosis, tuberculosis, and* M. sherrisii*. So, to our knowledge, this is the first detailed case report of infection by* M. sherrisii* in a patient with pneumoconiosis and history of tuberculosis and the first case described in Brazil. Bensi et al. [16] described the isolation of* M. sherrisii* from a single sputum sample in a large Brazilian university hospital, with no correlation to clinical or radiological aspects.

Before the isolation of* M. sherrisii*, an antimicrobial scheme was initiated with streptomycin, ethambutol, rifampin, and clarithromycin to cover* M. avium*, the main NTM to infect patients with chronic lung disease in our community.

There are no consensus recommendations for the treatment of* M. sherrisii*. Reports on susceptibility testing showed sensitivity to clarithromycin, rifabutin, and sulfamethoxazole, as well as variable sensitivity to moxifloxacin, isoniazid, ethambutol, streptomycin, and amikacin. The isolates showed resistance to rifampin, ciprofloxacin, ofloxacin, and linezolid [4, 7, 8, 13–15]. With a positive culture, the treatment regimen was adjusted to replace rifampin with rifabutin and to introduce sulfamethoxazole. Unfortunately, the isolates obtained from our patient were not subjected to sensitivity testing. After two months of therapy, and until the last visit, the sputum cultures remained negative. It is our plan to complete 12 months of treatment, as of the first negative culture, based on general recommendations of the American Thorax Society/Infectious Diseases Society of America [2], even though such recommendations might not be appropriate for every patient and every NTM species [17].

*M. sherrisii* should be considered a possible etiological agent of lung infections in patients with pneumoconiosis and a history of tuberculosis. More data are needed to determine the best therapeutic scheme and the real importance of this pathogen in the long term.
